# Malaria in indigenous and non-indigenous patients aged under 15 years between 2007-2018, Amazonas state, Brazil

**DOI:** 10.1590/0037-8682-0617-2021

**Published:** 2022-10-21

**Authors:** Mateus Ferreira de Aguiar, Bruna Martins Meireles, Wuelton Marcelo Monteiro, Maria Jacirema Ferreira Gonçalves

**Affiliations:** 1 Universidade Federal do Amazonas, Escola de Enfermagem de Manaus, Manaus, AM, Brasil.; 2 Fundação de Medicina Tropical Dr. Heitor Vieira Dourado, Manaus, AM, Brasil.; 3 Universidade do Estado do Amazonas, Manaus, AM, Brasil.

**Keywords:** Malaria, Indigenous health, Associated factors

## Abstract

**Background:**

Malaria is a serious problem in children because the immune system is less developed, thus, causing more severe symptoms. This study aimed to identify factors associated with malaria in indigenous and non-indigenous patients aged under 15 years in Amazonas, Brazil, from 2007 to 2018.

**Methods:**

An epidemiological, quantitative, cross-sectional study was conducted. Cases included patients aged under 15 years, using data from health system notifications between 2007 and 2018 in the state of Amazonas, Brazil. The variables included clinical-epidemiological, laboratory findings, and monitoring of cases. The outcome was ethnicity: indigenous, non-indigenous, and entries for which no ethnicity data were provided. A multivariable logistic regression model was used to compare the indigenous and non-indigenous populations.

**Results:**

Among malaria cases in patients aged under 15 years, there was a greater chance of being indigenous and having the following associated factors: female sex, children aged 0-4 years, passive case surveillance, a high load of parasitemia and the lack of data regarding the level of parasitemia, *Plasmodium falciparum* infections were more frequent, and timeliness of treatment, i.e., the interval between the onset of symptoms and time of treatment was within 48 hours.

**Conclusions:**

The factors associated with malaria are more frequent in indigenous populations and highlight differences according to ethnicity, suggesting that the severity of the disease is attributable to the increased number of malarial infections within this population. As a result, malaria has a greater impact on the health of indigenous people.

## INTRODUCTION

Malaria is an infectious disease caused by protozoa belonging to the genus *Plasmodium* and is transmitted to humans by female mosquitoes of the genus *Anopheles*. Two species of parasites are important for public health in Brazil: *Plasmodium vivax*, which sometimes causes mild symptoms, and *P. falciparum*, which has a greater potential for malaria-related deaths^1^.

In Brazil, the epidemiological situation of malaria is most severe in the Amazon region, mainly when analyzed in incident cases, as almost all cases are registered in this part of the country^1^. The disease transmission is greatly associated with forest areas or areas in proximity to forests, as these regions are the natural habitat of the *Anopheles darlingi* vector^1^. Therefore, indigenous populations present greater vulnerability than that of the general population, either of their habitat or cultural way of living^2^. In addition, although there has been an expansion of indigenous health care in Brazil, access to health facilities is still hampered, either by the geographic isolation of remote areas or the shortage of professionals available to work in these areas^3^. Furthermore, other factors are being identified as malaria-associated regardless of ethnicities (indigenous or non-indigenous), such as socio-economic and environmental issues, mining and mineral prospection areas, migratory and housing conditions, and occupational activities, which are directly linked to increased malaria transmission in the Amazon region^4^. In 2017, the Legal Amazon reported 99% of malaria cases in the country, with 42% of these cases registered in the state of Amazonas^5^. Between 2019 and 2020, there was a 15.2% increase in malaria cases among the indigenous people in Brazil^6^. 

Although the disease affects all age groups, it is a greater threat to children considering the following factors: the potential of the disease to deteriorate health, its difficult assessment as most clinical symptoms are non-specific, and most cases occur in places where routine malaria tests are not readily available^7^. As such, children aged under five years are particularly susceptible to infection, disease, or death. More than two-thirds of malaria-related deaths occurred in this age group^8^.

Malaria does not endanger a person’s health during the active phase of the disease. However, the consequences of malaria interfere with the quality of life and productivity of the population and, in children, can jeopardize development and learning^9^. In addition, other factors, such as iron deficiency, malnutrition, and intestinal parasitoses^10^ ^-^ ^11^ increase the degree of symptoms in this pediatric age group. 

Studies have pointed to the severity and poor care of indigenous children and adolescents, who were the main victims of the malaria outbreak that hit the Upper Negro River in 2018. It affects 60% of this population group^12^, with a higher incidence than that of the non-indigenous populations. Thus, this study aimed to identify factors associated with malaria in indigenous and non-indigenous patients aged under 15 years in Amazonas, Brazil, from 2007 to 2018.

## METHODS

### Study type

This is a cross-sectional epidemiological study that used secondary data from the Information System for Epidemiological Surveillance of Malaria (SIVEP-Malaria). The retrieved data included children and adolescents aged under 15 years with malaria between 2007 and 2018, and when the state of Amazonas was reported as a place of residence and probable infection setting.

This age group was selected because the available data were stratified by age group and not by age. Nevertheless, we did not analyze mortality, and we included children and adolescents aged under 15 years, regardless of whether this is mixed or not using only children, as children have been used in other research^13^ ^-^ ^14^. Children aged under five years contribute to the majority of reported cases; on the other hand, older children often experience low-density asymptomatic infections and have been identified as important contributors to the infection reservoir for onward transmission^14^.

### Study population and data source

All malaria cases involving patients aged under 15 years who reported to the SIVEP-Malaria between 2007 and 2018 were included in the study population. Sampling criteria were not considered, and all records were included. Regarding the inclusion criteria, all Amazonas residents with the same state as a probable site of infection were considered. Therefore, imported cases were excluded from the analysis.

It is important to note that since these are secondary data obtained without the identification of the persons, records only reflect episodes of malaria and not the patient itself. In view of this, there may be people with more than one episode of malaria within the study period and thus are included in the study. In addition, if the registers were “cure check slides”, they were excluded as they had already been reported as a new case and had a previous entry in the database.

### Organization of data

The SIVEP-Malaria data were made available by the Amazonas Health Surveillance Foundation (FVS-AM), obtained in an Excel spreadsheet format, and analyzed using Stata^®^ 13.0 software (StataCorp). These data were reviewed to separate indigenous and non-indigenous, and entries for which no ethnicity information was given. The samples were then filtered again to identify the cases for which Amazonas was given a place of residence and probable place of infection.

For the definition of “indigenous,” the criterion of another study on indigenous people in Amazonas, Brazil, was adopted due to the high number of entries with missing data in the “ethnicity” field, which represented 51.4% of the notifications in the study period^15^. The ethnicity field was included in the notification form in 2013, and it was not mandatory to fill in this information. To deal with this problem, a person was considered indigenous if the data already mentioned the information on ethnicity; in the other cases, we sought to identify whether the place of probable infection was given as “indigenous village”, if so, “indigenous” was assigned. Despite this limitation, a similar strategy has been used in other research^16^ ^-^ ^17^. It was determined that if the place of infection was an indigenous village, it could be assumed that patients aged under 15 years would be indigenous because there would be very few cases of non-indigenous patients aged under 15 years living in an indigenous village^18^. 

### Study variables

The variables used in the analyses were grouped as following:

- Clinical-epidemiological and laboratory findings were as follows: sex (male or female); age group (0-4, 5-9, and 10-14); type of surveillance slide (active or passive case surveillance); parasitemia level (<+/2 [at less than half a cross], +/2 [half a cross], + [one cross], ++ [two crosses], +++ [three crosses], and ++++ [four crosses]); test results (1- *P. falciparum* [*P. falciparum, P. falciparum* and gametocytes*,* and/or *P. falciparum* gametocytes]; 2- *P. vivax*; and 3- mixed [*P. vivax* and *P. falciparum* gametocytes, and/or *P. falciparum* + *P. vivax*], and others, including *P. malariae* and *P. ovale;*
- Monitoring of malaria cases: the interval between first symptoms and diagnosis (days), the interval between first symptoms and treatment (days), and treatment opportunity (relationship between date of diagnosis and start of treatment), which was considered early when it was less than 48 hours.

### Data analysis

For the descriptive phase, data were analyzed according to frequency and distribution and presented as percentages for categorical variables. 

In the analytical phase, we performed a bivariable analysis using Pearson’s chi-square test. The effect size was evaluated using Cramer’s V coefficient, including indigenous, non-indigenous, and missing data on ethnicity (missing data) as a comparison factor. We chose to display the missing data to present an overview of the situation and test in the multivariable analysis to check whether the lack of data would alter the association of malaria in indigenous patients aged under 15 years. 

We calculated the standardized residuals of the chi-square test among the categorical variables, which allowed us to identify the characteristic patterns in each category of a variable according to the excess or lack of occurrence of the combination of each category of the other variables. This allowed us to understand the significance of the associations, especially within the category. The residuals of the chi-square test with positive values greater than 1.96 corresponded to a level of significance of 5% and 95% confidence interval (CI)^19^.

For multivariable analysis, logistic regression adjusted in stepwise forward was used, and the outcomes were indigenous and non-indigenous. In the modeling process, the < 0.10 criterion of the significance of the *P*-value was used for inclusion in the model from the bivariable analysis. Variables with statistical significance (*P* < 0.05) were retained in the final model, and the results were reported using odds ratios (OR) together with their 95% CI. Model fit was evaluated using the Hosmer-Lemeshow test^20^.

### Ethical Aspects

As the data used had already been registered in the SIVEP-Malaria system and the ethnicity classification was completed at the time of notification of the case, there was no need for consent because the authors did not contact the patients, and the data were made available without identification. The Research Ethics Committee approved the project at the Federal University of Amazonas under protocol number 2.302.738.

## RESULTS

The results are presented in tables containing the characteristics of the patients (Table 1), clinical and laboratory aspects (Table 2), case monitoring (Table 3), and factors associated with malaria in indigenous patients aged under 15 years (Table 4). A total of 1,031,756 malaria cases were registered in the SIVEP-Malaria system, with probable infection and residence in the state of Amazonas, Brazil. Patients with registration errors in age and sex, as well as in the cure check slide, were excluded. Thus, 938,714 cases remained from 2007 to 2018. Children and adolescents aged under 15 years accounted for 370,603 of these cases, of which 51.4% were missing data (without information on ethnicity). In the cases of patients aged under 15 years with data on ethnicity, 23.4% were indigenous, and 25.1% were non-indigenous. 

**TABLE 1:** Malaria cases in patients aged under 15 years according to indigenous, non-indigenous, and missing data, Amazonas state, Brazil, 2007-2018.

Variable	Indigenous*		Non-indigenous		Missing data		P-value
**Age group (years)**	N = 86,841	%	N = 93,191	%	N = 190,571	%	(Pearson’s chi-square test)
	(SR)		(SR)		(SR)		
Under 1 year	4,538	5.23	3,151	3.38	7,255	3.81	(< 0.01)
	**(20.42)**		(-11.67)		(-7.17)		Cramer’s V = 0.07
1 to 4	29,989	34.53	23,422	25.15	55,275	29.00	
	**(38.51)**		(-32.50)		(-4.42)		
5 to 9	29,850	34.37	31,517	33.81	65,859	34.56	
	(0.31)		(-3.78)		(3.02)		
10 to 14	22,464	25.87	35,101	37.66	62,182	32.63	
	(-46.40)		**(40.39)**		(4.25)		
**Sex**							(Pearson’s chi-square test)
Female	41,610	47.92	41,969	45.04	87,942	46.15	(< 0.01)
	**(11.03)**		(-8.82)		(-1.69)		Cramer’s V = 0.02
Male	45,231	52.08	51,221	54.96	102,629	53.85	
	(-11.03)		**(8.82)**		(1.69)		

**Source:** Information System for Epidemiological Surveillance of Malaria (SIVEP-Malaria), data obtained in December 2019.

**Notes: SR:** standardized residual. The percentage is shown in the column. In bold are the residuals of the chi-square with a positive value greater than 1.96, which corresponds to the level of significance for excess occurrences. *Indigenous is the combination of the individual with race declared indigenous or having the tribal village as the place of infection.

**TABLE 2:** Clinical and laboratory aspects of malaria in patients aged under 15 years according to indigenous, non-indigenous, and missing data, Amazonas state, Brazil, 2007-2018.

Variable	Indigenous*		Non-indigenous		Missing data		P-value
**Type of surveillance slide**	N = 86,841	%	N = 93,191	%	N = 190,571	%	(Pearson’s chi-square test)
	(SR)		(SR)		(SR)		
Active case surveillance	40,693	46.86	58,777	63.07	122,426	64.24	(< 0.01)
	**(-89.42)**		**(23.01)**		**(55.81)**		Cramer’s V = 0.14
Passive case surveillance	46,148	53.14	34,414	36.93	68,145	35.76	
	**(89.42)**		(-23.01)		(-55.81)		
Parasitemia level by plus system scale							(Pearson’s chi-square test)
Up to 1 + (up to 10 parasites per 100 thick film fields)	55,272	63.65	62,798	67.39	171,372	71.23	(< 0.01)
	(-6.77)		**(20.49)**		(-12.05)		Cramer’s V = 0.07
++ (11 to 100 parasites per 100 thick film fields)	27,001	31.09	27,809	29.84	63,017	26.19	
	(-5.06)		(-14.79)		**(17.13)**		
+++ or more (> 100 parasites per one thick film field)	2,725	3.14	1,953	2.10	6,181	2.57	
	**(4.15)**		(-17.45)		**(11.63)**		
Number of crosses (+) not filled in	1,843	2.12	631	0.68	1	0.01	
	**(60.13)**		(0.40)		(-51.31)		
Test result							(Pearson’s chi-square test)
*Plasmodium falciparum* (F, F+FG, FG)	10,891	12.54	7,209	7.73	23,630	12.40	(< 0.01)
	**(13.65)**		(-39.34)		**(22.57)**		Cramer’s V = 0.05
*Plasmodium vivax*	74,959	86.32	85,634	91.89	165,486	86.83	
	(-17.28)		**(42.37)**		(-22.13)		
Mixed (F+V, V+FG)	984	1.13	345	0.37	1,454	0.76	
	**(14.90)**		(-15.56)		(0.87)		
Others (including *P. malariae* and *P. ovale*)	7	0.01	3	0.01	1	0.01	
	**(3.14)**		(0.16)		(-2.81)		

Source: Information System for Epidemiological Surveillance of Malaria (SIVEP-Malaria), data obtained in December 2019.

Notes: SR : standardized residual. The percentage is shown in the column. In bold are the residuals of the chi-square with a positive value greater than 1.96, which corresponds to the level of significance for excess occurrences. Test results: F: *Plasmodium falciparum*; FG: *Plasmodium falciparum* gametocytes; V: *Plasmodium vivax.* *Indigenous is the combination of the individual with race declared indigenous or having the tribal village as the place of infection.

**TABLE 3:** Timeliness of diagnosis and treatment of malaria in patients aged under 15 years according to indigenous, non-indigenous, and missing data, Amazonas state, Brazil, 2007-2018.

Variable	Indigenous*		Non-indigenous		Missing data		P-value
**Days between the first symptoms and the diagnosis**	N = 86,841	%	N = 93,191	%	N = 190,571	%	(Pearson’s chi-square test)
	(SR)		(SR)		(SR)		
0	22,542	25.96	15,185	16.30	35,882	18.83	(< 0.01)
	**(51.46)**		(-31.45)		(-16.23)		Cramer’s V = 0.07
1	17,927	20.65	22,175	23.80	44,739	23.48	
	(-18.02)		**(7.60)**		**(8.69)**		
2	14,118	16.26	18,816	20.19	36,376	19.09	
	(-21.11)		**(13.48)**		**(6.20)**		
3	9,117	10.50	12,346	13.25	25,079	13.16	
	(-20.93)		**(7.35)**		**(11.37)**		
4 to 7	10,403	11.98	13,163	14.13	28,154	14.77	
	(-19.21)		**(1.73)**		**(14.78)**		
> 7	12,724	14.65	11,493	12.33	20,325	10.67	
	**(27.27)**		**(3.41)**		(-26.07)		
**Days between diagnosis and treatment**							(Pearson’s chi-square test)
0	18,899	21.77	13,717	14.72	28,814	15.12	(< 0.01)
	**(46.98)**		(-17.60)		(-24.53)		Cramer’s V = 0.06
1	17,784	20.48	21,761	23.35	42,185	22.14	
	(-12.79)		**(11.06)**		(1.24)		
2	14,686	16.91	18,920	20.31	35,553	18.66	
	(-15.12)		**(14.88)**		(-0.10)		
3	9,777	11.26	12,657	13.58	25212	13.23	
	(-16.07)		**(7.66)**		**(6.98)**		
4 to 7	11,874	13.67	14,011	15.04	29,901	15.69	
	(-12.99)		(-0.17)		**(11.15)**		
> 7	13,812	15.91	12,110	13.00	28,897	15.16	
	**(10.56)**		(-17.85)		**(6.54)**		

**Source:** Information System for Epidemiological Surveillance of Malaria (SIVEP-Malaria), data obtained in December 2019. **Notes: SR:** standardized residual. The percentage is shown in the column. In bold are the residuals of the chi-square with a positive value greater than 1.96, which corresponds to the level of significance for excess occurrences. *Indigenous is the combination of the individual with race declared indigenous or having the tribal village as the place of infection.

**TABLE 4:** Crude and adjusted odds ratio of malaria-associated factors in indigenous patients aged under 15 years, Amazonas state, Brazil, 2007-2018.

Variables	%	Crude OR	95% CI	Adjusted OR	95% CI
**Sex**					
Male	52.08	1		1	
Female	47.92	1.12	1.10-1.14	1.12	1.10-1.14
**Age group (years)**					
Under 1 year	5.23	2.25	2.14-2.36	2.20	2.09-2.31
1 to 4	34.37	2.00	1.95-2.04	1.95	1.90-2.20
5 to 9	25.87	1.48	1.44-1.51	1.43	1.40-1.46
10 to 14	25.87	1		1	
**Type of surveillance slide**					
Active case surveillance	46.86	1		1	
Passive case surveillance	53.14	1.94	1.9-1.97	1.81	1.77-1.84
**Parasitemia level by plus system scale**					
Up to 1 + (up to 10 parasites per 100 thick film fields)	63.65	1		1	
++ (11 to 100 parasites per 100 thick film fields)	31.09	1.10	1.08-1.12	1.17	1.14-1.19
+++ or more (> 100 parasites per one thick film field)	3.14	1.58	1.49-1.68	1.54	1.44-1.63
Number of crosses (+) not filled in	2.12	3.31	3.02-3.63	3.07	2.79-3.39
**Test result**					
*Plasmodium vivax*	12.54	1		1	
*P. falciparum* (F, F+FG, FG)	86.32	1.72	1.67-1.78	1.71	1.65-1.76
Mixed (F+V, V+FG)	1.13	3.26	2.88-3.68	2.74	2.42-3.11
**Timeliness of treatment (hours)**					
> 48 h		1		1	
≤ 48 h		2.31	2.23-2.38	2.05	1.98-2.12

Source: Information System for Epidemiological Surveillance of Malaria (SIVEP-Malaria), data obtained in December 2019. Notes: OR: odds ratio; 95% CI: 95% confidence interval. Significance level < 0.10, crude analysis; significance level < 0.05, adjusted analysis. Timeliness of treatment: time from symptom onset to treatment initiation. Test results: F: *Plasmodium falciparum*; FG: *Plasmodium falciparum* gametocytes; V: *Plasmodium vivax*.

Table 1 shows the distribution of reported cases of malaria in patients aged under 15 years by sex and age group, according to ethnicity: indigenous or non-indigenous, and missing data (without ethnicity information). Standardized residuals show an excess occurrence in indigenous patients for females and children aged under one year, while in non-indigenous patients, the excess occurrence was for males and age group 10 to 14 years. The effect size was estimated to be small, as revealed using Cramer’s V test (0.07 and 0.02, for age group and sex, respectively), with statistically significant *P*-values (< 0.01). Regarding the indigenous age group, there was a higher frequency of malaria in children aged under five years, whose proportion decreased as the age group increased, in contrast to what occurs in non-indigenous and in cases of missing data for ethnicity (Table 1). 

Table 2 presents the clinical and laboratory data of the reported cases of malaria in patients aged under 15 years, according to indigenous, non-indigenous, and missing data (without ethnicity information). Despite the small effect size observed using Cramer’s V test for the type of surveillance slide (V = 0.14), parasitemia (V = 0.07), and test results of the infectious agent (V = 0.05), the following highest frequencies were noted, with statistically significant *P*-values (< 0.01) in the standardized residuals of the chi-square test: (a) in non-indigenous - active case surveillance, lower parasitic load (up to one cross), and *P. vivax* infection; (b) in cases lacking information on ethnicity (missing data) - active case surveillance, two or more crosses of parasitic load, and disease caused by some stages form of *P. falciparum* parasite; (c) and in indigenous - passive case surveillance, three or more crosses of parasitic load or missing data in the crosses number field, and disease caused by some stages form of *P. falciparum* parasite, *P. vivax,* or other parasites.

The association between the number of days between the date of onset of symptoms and diagnosis and ethnicity, as well as the time between diagnosis and the start of treatment and ethnicity, were found to be small in terms of effect size evaluated using Cramer's V test (0.07 and 0.06, respectively), with statistically significant *P*-values (< 0.001), as shown in Table 3.

Regarding the number of days between the date of onset of symptoms and diagnosis, the excess occurrence was 0 days and more than 7 days in the indigenous populations. The non-indigenous populations showed excess occurrence for 1, 2, 3, and > 7 days. The missing data on ethnicity presented significant residuals of the chi-square test for either 1 to 7 days, indicating no distinction for early diagnosis in this category. Regarding the time between diagnosis and the start of treatment, the result was similar to the days between the first symptoms and treatment, except for cases in which there was no data on ethnicity, with significance in the residuals of the chi-square test for 3 days or more (Table 3).

The results of the multivariable logistic regression analysis, which estimated the factors associated with malaria cases in indigenous and non-indigenous patients aged under 15 years, with the respective values of crude and adjusted ORs, are presented in Table 4. To compare malaria cases involving indigenous or non-indigenous, data without the ethnicity information were excluded from the analysis. As shown in Tables 1 to 3, cases of missing data can be excluded as they do not affect the comparison, nor do they present divergence between associated factors in indigenous and non-indigenous malaria reported cases in children and adolescents aged under 15 years.

All variables tested in the bivariate logistic analysis were statistically significant for inclusion in multivariate analysis. In the final model, we highlight the factors associated with malaria in indigenous compared with those non-indigenous children and adolescents, which presented the greatest magnitude: female sex (OR = 1.12; 95% CI 1.10-1.14); age group younger than 1 year (OR = 2.2; 95% CI 2.09-2.31), with a reduction of the OR as age increases, maintaining statistical significance; passive case surveillance (OR = 1.81; 95% CI 1.77-1.84); a high load of parasitemia (OR = 1.54; 95% CI 1.44-1.63) and the lack of information on parasitic load field (OR = 3.07; 95% CI 2.79-3.39); mixed parasitic infections (F+V, V+FG) (OR = 2.74; 95% CI 2.42-3.11); timeliness of treatment, i.e., the start of treatment within 48 h from the date of the first symptoms (OR = 2.05; 05% CI 1.98-2.12) (Table 4).

## DISCUSSION

The factors associated with malaria among reported cases of children and adolescents warrant attention and proper care in this population group. The differences observed between indigenous and non-indigenous patients aged under 15 years indicate that the disease affects ethnic groups in different ways.

The association between malaria in patients aged under 15 years and female sex is an enigma; such that one would not expect sex to have a great significance despite the predominance of male sex in the study population, nor a difference in the association between non-indigenous and that of which is already known in adults^21^. Furthermore, no other studies have identified an association between malaria and female sex in indigenous children and adolescents. 

Detecting a stronger association of malaria in the younger age group is an indicator of the severity of the problem in this population. Children aged under 1 year have an immature immune system, making them more vulnerable to infectious agents^22^. Because malaria affects children early, there is a risk that the sequelae of the disease will also affect this population more strongly, in addition to the risk of multiple malarial diseases accumulated throughout life, since the first experiences occur at an early age. From a clinical and child development perspective, malaria has a combination of biological determinants, such as immunity, as well as cultural determinants in the form of housing, coexistence in society, and socio-political aspects in relation to access to health services. Therefore, these elements must contribute to malaria in indigenous areas, as they present differentiated epidemiological behavior^16^. 

Malaria is widely distributed in non-indigenous people aged 10 to 14 years and may be related to intra-and peridomicile transmission if we consider housing conditions and proximity to forest areas, vector density, or basic sanitation^23^. In addition, the responsibility of helping parents with their quotidian tasks, such as farming, fishing, hunting, and agriculture, often results in a situation where children and adolescents are exposed to proximity to the vector, which can contribute to infection rates.

Indigenous people have specific health policies that the National Health Service (Sistema Único de Saúde - SUS) should adapt to and consider when creating organizational models that aim to promote health and prevent the spread of malaria for this group^2^. In addition, some characteristics, such as the high mobility of indigenous people, difficulty of accessing their regions by health teams, and persistent incursion of gold miners that hinders malaria control interventions in these regions, contribute to the change in the epidemiological profile of the disease^24^.

In indigenous populations, among the reported malaria cases, passive case surveillance is more frequent for cases of infection in children aged under five years, possibly due to mothers taking their children to healthcare facilities as soon as their first symptoms appear. In contrast, in non-indigenous populations, active case surveillance with high occurrence demonstrates the efficiency of investments and efforts established to ensure an increased network of malaria laboratories throughout the Amazonas state, with an increment of 72% in 10 years^25^. However, even with the incentive for the development of rapid tests in Brazil, especially in regions away from large centers, passive case surveillance occurs more frequently in indigenous cases^13^. Moreover, healthcare systems in indigenous areas of Brazil are marked by a lack of health professionals and difficulties faced by the population in accessing these services^3^

One of the most commonly used techniques for the laboratory diagnosis of malaria is a thick blood smear that seeks specific confirmation of the disease. This technique is important because it allows visualization of parasites, species identification, and stages of development and quantification, which are essential data for clinical evaluation^26^. A thick blood smear is a sensitive method capable of detecting 0.001% of parasitemia. However, the method becomes less sensitive when the individual is infected with more than one species of *Plasmodium*; 29% of infections diagnosed by thick blood smear are that of *P. vivax*, when analyzed by polymerase chain reaction (PCR), were identified as mixed infections^27^. In the SIVEP-Malaria system, parasitemia was quantified using a plus system scale and presented in crosses to facilitate presentation and explanation. In this format, indigenous people have a greater parasitic load than that of non-indigenous people. This demonstrates that because they live in places more vulnerable to mosquito breeding sites, they are more exposed to bites, or their immune system is not as competent in containing the multiplication of parasites after infection^4^. In addition, the lack or difficulties in health access must be pointed out to obtain a diagnosis and treatment with antimalarial drugs^3^.

Regarding the *Plasmodium* species present, *P. vivax* was shown to be an infectious agent prevalent in non-indigenous populations, which is similar to another study^27^. This trend can be explained by factors such as its wide geographical distribution since *P. vivax* establishes itself under higher temperature conditions^28^ and is characterized by more comprehensive and sometimes insidious symptoms, such as fever, headache, and chills. Despite the indigenous ethnic group having the highest percentage of *P. falciparum*, the most common parasite was *P. vivax*.

The efforts of the program soon led to a reduction in *P. falciparum* cases, even though the total number of cases remained relatively high. Hyperendemic transmission is thought to be affected by extensive anthropogenic environmental changes in conjunction with the way of indigenous life in the region, which are likely to have extended the habitats and increased the density of vectors, rendering vector control tools insufficient^1^.

It is worth noting that cases of *P. falciparum* and mixed malaria (*P. falciparum* and *P. vivax*) are predominant in indigenous people, as they are more severe in these cases. However, as symptoms exacerbate, this contributes to the immediate search for a diagnosis since passive malaria case surveillance has the largest percentage among indigenous people. In addition, indigenous people aged under 15 years have diverse parasitic forms of infection, with a prevalence of mixed infections (*P. falciparum* plus *P. vivax* and *P. vivax* and *P. falciparum* gametocytes).

The high proportion of *P. falciparum* infection is extremely alarming, especially if people live in isolated areas, where traditional health system practices are largely unavailable, and remains a considerable challenge for case detection and proper follow-up. It is important to note that the literature has revealed the possibility of vulnerability arising from the Duffy-negative (Fy-) blood group, leading to the predominance of *P. falciparum.* However, this aspect was not investigated in this study^13^.

The timeliness of treatment of malaria among indigenous people in less than 48 hours meets the recommendations set by the Brazilian Ministry of Health and is an important factor for the control of the disease; the earlier the treatment, the lower the possibility of spread by reducing the source of infection. It is also an indicator of the level of care provided because in the context studied, indigenous children and adolescents had both diagnosis and received treatment earlier compared with those non-indigenous. It may also be attributed to the prompt care they receive as soon as the first symptoms appear and the immediate pursuit of health services where diagnosis and treatment are provided. It is also possible that it is related to the delivery of health services in indigenous areas, in which access is facilitated due to close proximity to the area. For example, the person who usually performs the thick blood smear examination is a professional living in the area or even an indigenous professional^5^.

The data presented by the SIVEP-Malaria system, like any secondary data, presents potential biases that are out of the researchers’ control, such as the lack of standardization in data collection, which affects the quality of the records, the coverage that can vary in time and space, and lack of information, as in the case of the variable ethnicity and parasitemia, both showing a high percentage of lack of data. In the first case, it was demonstrated that the classification method used in this work, including the indigenous village as a place of probable infection, contributed to the increase in ethnicity data completeness. However, in the second case, the lack of quantitative information about parasitemia was used in the analysis, thus, limiting the interpretation of the parasitic burden. For missing parasitic data, there is a possibility of both high and low parasite loads because both make counting difficult and require greater microscopist skill, which may be an additional vulnerability in indigenous areas. Although these limitations have not compromised the interpretations of this study, additional analyses are necessary to unravel both the epidemiological situation of malaria involving indigenous people and the effectiveness of the actions focused on indigenous health aimed at the prevention and control of endemic malaria. 

The factors associated with malaria in indigenous children in Amazonas may indicate that the disease is facilitated by their way of life and the organization of health services in that region. Understanding these factors contributes to the development of strategies to provide proper healthcare to populations who are often vulnerable due to geographical, economic, or biological reasons.

The results suggest that actions appropriate to the peculiarity of this population need to be developed to reduce the incidence of malaria in children and adolescents, especially among those aged under 5 years; thus, this group may not experience further harm, such as the likelihood of repeated infections when malaria is acquired early. Therefore, malaria control programs should be implemented to improve efforts to contain the disease in these groups. Furthermore, the results of this research may guide the delineation of more effective and assertive decisions by professionals responsible for reducing *Plasmodium* infection in these areas. 
